# Supplementary material for: Probing spin dynamics of ultra-thin van der Waals magnets via photon-magnon coupling
Source: Nat Commun. 2023 May 5;14:2619. doi: 10.1038/s41467-023-38322-x (PMC10163026; doi:10.1038/s41467-023-38322-x)
Supplement: Supplementary file 1 — Supplementary Information [file 41467_2023_38322_MOESM1_ESM.pdf]

Supplemental Material - Probing spin dynamics of ultra-thin van der Waals magnets via photon-magnon coupling

Christoph W. Zollitsch,[1,a)] Safe Khan,[1] Vu Thanh Trung Nam,[2] Ivan A. Verzhbitskiy,[2] Dimitrios Sagkovits,[1,3] James O'Sullivan,[1] Oscar W. Kennedy,[1] Mara Strungaru,[4] Elton J. G. Santos,[4,5] John J. L. Morton,[1,6] Goki Eda,[7,2,8] and Hidekazu Kurebayashi[1,6,9]

[1)] *London Centre for Nanotechnology, University College London, 17-19 Gordon Street, London, WCH1 0AH, UK*
[2)] *Department of Physics, Faculty of Science, National University of Singapore, 2 Science Drive 3, Singapore 117542, Singapore*
[3)] *National Physical Laboratory, Hampton Road, Teddington TW11 0LW, UK*
[4)] *Institute for Condensed Matter Physics and Complex Systems, School of Physics and Astronomy, The University of Edinburgh, Edinburgh EH9 3FD, UK*
[5)] *Higgs Centre for Theoretical Physics, The University of Edinburgh, Edinburgh EH9 3FD, UK*
[6)] *Department of Electronic & Electrical Engineering, UCL, London WC1E 7JE, United Kingdom*
[7)] *Centre for Advanced 2D Materials, National University of Singapore, 6 Science Drive 2, Singapore 117546, Singapore*
[8)] *Department of Chemistry, Faculty of Science, National University of Singapore, 3 Science Drive 3, Singapore 117543, Singapore*
[9)] *WPI Advanced Institute for Materials Research, Tohoku University, 2-1-1, Katahira, Sendai, 980- 8577, Japan*

(Dated: 25 April 2023)

I. MICROWAVE SETUP AND MEASUREMENT

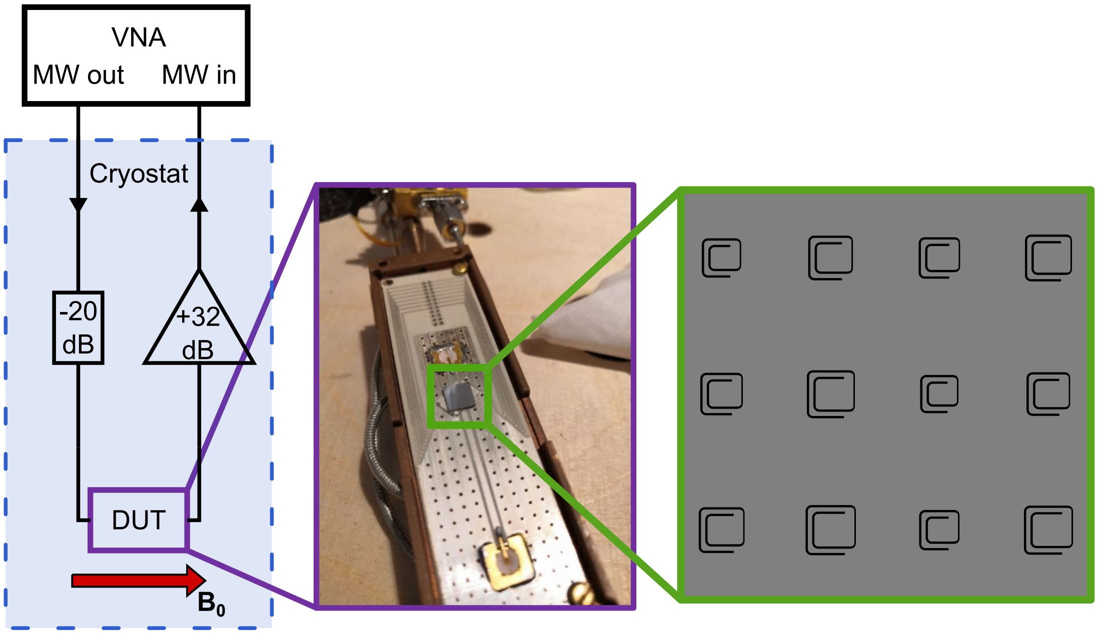

FIG. S1. **Microwave delivery and detection setup.** Schematic of the microwave delivery and detection circuit. The image shows the coplanar waveguide transmission line. A resonator chip is placed on top of the transmission line for read out. On the right, a schematic layout of the resonators on a single chip is shown.

[a)] Electronic mail: c.zollitsch@ucl.ac.uk

Figure S1 shows a schematic of the used microwave measurement setup. We are using a Keysight E5071C vector network analyzer (VNA) to deliver and detect microwaves. The VNA is connected to a low temperature probe, fitted into a closed cycle helium cryostat and cooled to a base temperature of about 1.8 K. The microwave signal is transmitted into the cryostat and is attenuated by −20 dB. The attenuator is positioned just before the sample box and provides a thermal anchoring for the center conductor of the coaxial cable to minimize the thermal load onto the sample. The output line is equipped with a Low Noise Factory LNC6_20C cryogenic amplifier, operating between 6 − 20 GHz with an average amplification of +32 dB. The transmitted and amplified signal is finally detected by the VNA. Figure S1 also shows an image of the coplanar waveguide transmission line PCB, loaded with a resonator ship, of which a schematic shows the resonator layout on a single chip. The resonators on the chip are capacitively coupled to the transmission line PCB. Upon resonance the transmission through the PCB is reduced, indicating the resonator resonance. The cryostat is equipped with a mechanical rotation stage and prior to the measurements the superconducting resonators are carefully aligned to the externally applied static magnetic field B_0, such that the field is in the plane of the superconductor and along the narrow section of the resonators.

Figure S2 shows the raw uncalibrated microwave transmission, ranging from 10 GHz to 18 GHz. The transmission is dominated by imperfections in our microwave circuitry, masking the small signals from the superconducting resonators. Thus, we performed a simple thru calibration of the microwave transmission to remove contributions from the setup, prior each magnetic field dependent measurement. Here, we exploit the magnetic field tunability of our superconducting resonators. Before calibration, we set the frequency range of the measurement. We change the applied magnetic field such that the resonator's resonance frequency is tuned out of the set frequency range. With a frequency window just showing the transmission of the setup we perform the thru calibration. After calibration we set the magnetic field back to its starting value, resulting in a background corrected spectrum with just the resonator feature on it.

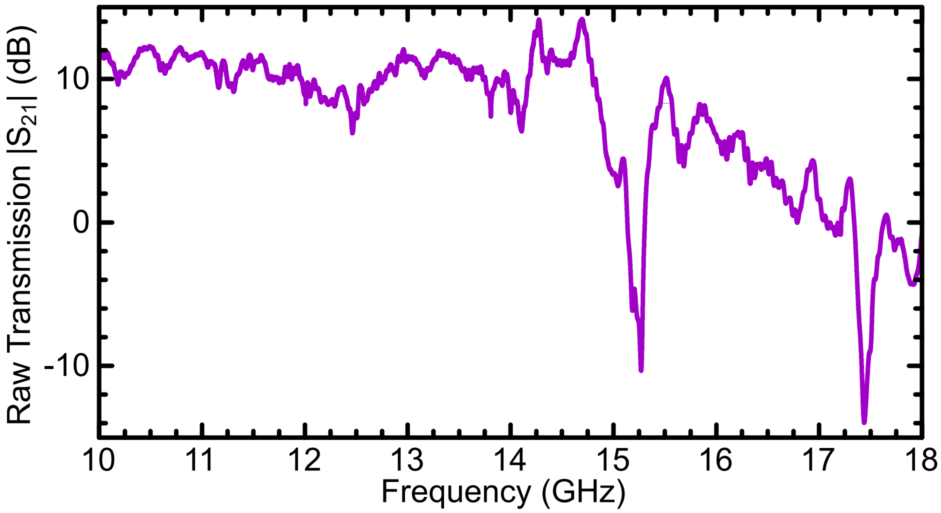

FIG. S2. **Raw broadband microwave transmission signal.** Logarithmic microwave transmission $|S_{21}|$ as a function of frequency between 10 GHz and 18 GHz at a temperature of 1.8 K.

II. RESONATOR CHARACTERIZATION

In this study, we fabricate twelve superconducting lumped element resonators on each of three resonator chips were fabricated using the same design (see schematic Fig. 1 (a) in the main text). Prior to transfer of the CGT flakes, we characterized the resonators at a temperature of 1.8 K and zero applied magnetic field, using microwave powers of about −80 dBm at the resonators, which is well below the bifurcation limit starting above −60 dBm. Due to finite fabrication tolerances the resonator parameters have some variation, while some didn't work at all. However, the targeted resonance frequencies are well reproducible and very similar for the 3 different chips. We compare the resonator parameters before and after transfer of the CGT flakes and collate the parameters in Tab. I. Note, the resonator parameters with the CGT flakes on were obtained with a static magnetic field applied in the plane of the superconductor, but far detuned from the CGT FMR. In addition, we add the respective thickness of the flake on each resonator, acquired from AFM measurements. Here, we give the values of the thickest region of a given flake on a resonator, as the thickest region will dominate the FMR signal. Due to the arbitrary shape of exfoliated flakes, some exhibit regions of different thickness, as seen e.g. in Fig. S5 (h) and (i).

TABLE I. Resonator Parameters

Chip Number	$\omega_{\mathrm{res,before}}$ (MHz)	$Q_{\mathrm{L,before}}$	$\omega_{\mathrm{res,after}}$ (MHz)	$Q_{\mathrm{L,after}}$	CGT Thickness (nm)
1	12165	1978	12063	5733	16.2 ± 1.3
1	13303	7357	13177	4950	-
1	13968	5575	13860	4679	49.4 ± 3.5
1	14184	6492	14048	5627	153.1 ± 23.3
1	16648	6606	16470	5021	23.5 ± 2.5
1	17431	3215	17237	6826	23.8 ± 6.4
1	17959	7595	17790	3963	26.2 ± 4.1
2	12285	360	12153	7135	49.1 ± 9.1
2	12669	3600	12548	6693	102.8 ± 5.6
2	12782	3448	12648	6557	105.9 ± 3.9
2	13393	4643	13244	4501	34.4 ± 4.1
2	13760	6858	13620	5488	95.9 ± 5.9
2	14395	9048	14201	4139	36.7 ± 4.3
2	16075	7283	-	-	-
2	17048	6541	16811	4241	75.5 ± 5.4
3	12043	6114	11899	6044	59.7 ± 32.8
3	12456	2716	12314	6938	11.4 ± 1.8
3	12996	5828	12848	4600	17 ± 0.8
3	13422	6517	13272	5461	89.8 ± 7.5
3	13719	6800	13582	6608	-
3	14238	9184	14064	5420	73.5 ± 8.4
3	15390	8680	15219	6030	30.5 ± 4.2
3	15821	2386	15604	4769	33.1 ± 9.9
3	16430	7518	16193	5780	30.1 ± 38.1
3	17308	6521	17054	5569	137.9 ± 3.4
3	18111	3542	17870	4643	50.2 ± 6.9

III. RESONATOR AND COUPLING SIMULATION

We use finite element and numerical simulations to optimize our resonator design. Key requirements of our resonators are a strong resilience to externally applied static magnetic fields and a small mode volume. To achieve a large field resilience we reduced the area of the resonator to minimize effects of the magnetic field on the superconducting film. Further, we designed the resonators such that they act as lumped element resonators. Here, the resonance frequency is given by the total capacitance and inductance of the structure, with $\omega_{\mathrm{res}} = 1/\sqrt{LC}$, analogues to a parallel LC circuit. This allows us to locally separate oscillating electric and magnetic fields and also to concentrate the magnetic fields in more confined regions, resulting in very small mode volumes. To verify the lumped element nature of our resonators we performed finite element simulations, using CST Microwave Studio. Figure S3 shows the resulting magnitude of the E-field (left side) and H-field (right side) distribution along the resonator structure for the resonator design producing the results shown in Fig. 2 in the main text. The E-field is concentrated along the parallel running wire sections, with its strength approaching zero along the narrow wire section. The opposite is the case for the H-field, where it is zero along the parallel wire sections and strongly concentrated along the narrow wire section. Note, that the H-field magnitude is homogeneous along the whole of the narrow wire section.

The CST Microwave Studio at hand allowed us a simulation with perfect electric conductors. This is sufficient to model the general electric and magnetic energy distributions and resonance frequencies, however, not to simulate the corresponding oscillating magnetic field distribution, created by a superconducting rectangular wire. To this end, we numerically solve the Biot-Savart law for a rectangular wire cross-section[S1], assuming a superconducting current distribution $\mathbf{J}_{\mathrm{x,z}}$[S2],

$$\mathbf{B}_{1,\mathrm{x,z}} = \frac{\mu_0}{2\pi}\int_{-w/2}^{w/2}\int_{-d/2}^{d/2}\frac{\mathbf{J}\times\mathbf{r}}{(x-x')^2+(z-z')^2}dx'dz', \tag{S1}$$

with the vectors as $\mathbf{J} = (0, J(x,z), 0)^T$ and $\mathbf{r} = (x-x', 0, z-z')^T$ and μ_0 being the magnetic constant. The integration is performed over the cross-section of the wire, of width w and thickness d. We define the wire cross-section in the x-z-plane, with w in x-direction and d in z-direction. The length of the wire is along the y-direction. For a superconducting wire, the current is not homogeneously distributed over the cross-section of the wire. Current is only flowing on the surface and is exponentially decaying towards the center of the wire. The characteristic length scale is given by the London penetration depth λ_{L}. We use the

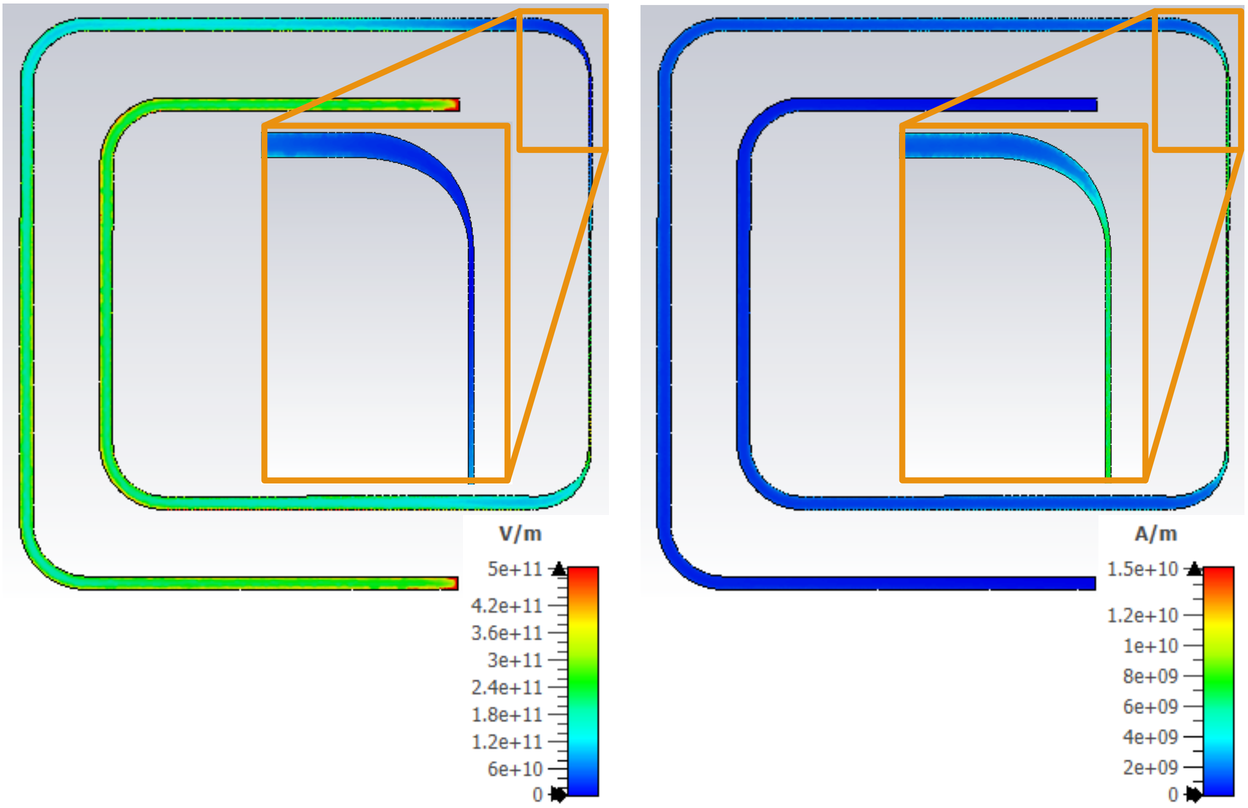

FIG. S3. **Finite element simulations of resonator.** CST Microwave Studio simulation of the distribution of E-fields and H-fields across the resonator structure. The color encoded fields represent the magnitude values.

following expression for the current distribution[S2]

$$J(x,z) = J_1 \left(\frac{\cosh{}^{z'}/_{\lambda_\mathrm{L}}}{\cosh{}^{d}/_{\lambda_\mathrm{L}}} \left[C \frac{\cosh{}^{x'}/_{l_1}}{\cosh{}^{w}/_{l_1}} + \frac{1 - \cosh{}^{x'}/_{l_2} / \cosh{}^{w}/_{l_2}}{\sqrt{1-({}^{x'}/_{w})^2}} \right] + \frac{J_2}{J_1} \frac{\cosh{}^{x'}/_{\lambda_\mathrm{L}}}{\cosh{}^{w}/_{\lambda_\mathrm{L}}} \right), \tag{S2}$$

where

$$\frac{J_2}{J_1} = \frac{1.008}{\cosh{}^{d}/_{\lambda_\mathrm{L}}} \sqrt{\frac{{}^{w}/_{\lambda_\perp}}{4 * {}^{\lambda_\perp}/_{\lambda_\mathrm{L}}} - 0.08301\,{}^{\lambda_\mathrm{L}}/_{\lambda_\perp}},$$

$$C = \left(0.506 \sqrt{{}^{w}/_{2\lambda_\perp}}\right)^{0.75},$$

$$l_1 = \lambda_\mathrm{L} \sqrt{{}^{2\lambda_\mathrm{L}}/_{\lambda_\perp}},$$

$$l_2 = 0.774\,{}^{\lambda_\mathrm{L}^2}/_{\lambda_\perp} + 0.5152\lambda_\perp,$$

$$\lambda_\perp = {}^{\lambda_\mathrm{L}}/_{2d}.$$

The prefactors J_1 and J_2 define the amplitude of the current density and hence the absolute value of the oscillating magnetic field B_1. We define J_1 by normalizing the vacuum B_1 field to the energy density stored in the resonator[S3,S4]

$$\frac{1}{2}\frac{\hbar\omega_\mathrm{res}}{2} = \frac{1}{2\mu_0} \int \mathbf{B}_1^2 dV = \frac{1}{2\mu_0} B_1^2 V_\mathrm{m}, \tag{S3}$$

with V_m representing the resonator mode volume. The additional factor of $^1/_2$ on the left hand side of S3 takes into account that only half of the total energy is stored in the magnetic field[S5]. As our resonator design is a quasi 1-dimensional structure we have to define boundaries for the mode volume in the x- and z-direction. A common assumption is to use the width of the conductor wire w[S6]. For simplicity, we approximate the x-z-area of the mode distribution with the area of an ellipse. For the last dimension we use the length of the narrow wire section, supported by the CST Microwave Studio simulations (see Fig. S3). In total we find

the mode volume to be $V_m = ((\pi 3.0\,\mu m \times 2.025\,\mu m) - w \times d) \times 300\,\mu m = 5696\,\mu m^3$. Figure S4 shows the resulting distribution of the oscillating magnetic field for the cross-section of the rectangular wire of width $w = 2\,\mu m$ and thickness $d = 50\,nm$. The magnitude $|\mathbf{B}_{1,x,z}|$ is encoded in the color and the arrows indicate the $B_{1,x}$ and $B_{1,z}$ components of the oscillating field.

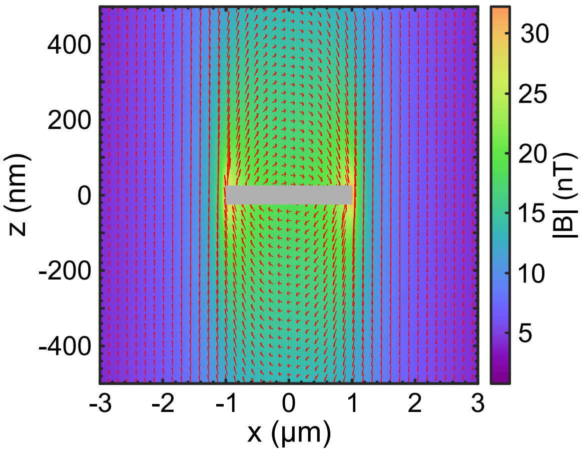

FIG. S4. **Cross-section of resonator magnetic field distribution.** Calculated magnitude of the magnetic field distribution around the cross-section of a rectangular superconducting wire. The wire cross-section lies in the center, indicated by the grey rectangular. The red arrows show the direction of the magnetic field.

With the simulated $\mathbf{B}_1$ field distribution we can calculate the position dependent single photon - single spin coupling strength $g_0(\mathbf{r})$[S3,S4] for each magnetic moment per unit cell of CGT (ab-plane 0.68 nm[S7,S8], along the c-axis 0.7 nm[S9]). Summation over all CGT unit cells N within the mode volume of the resonator results in the collective coupling strength

$$g_{\mathrm{eff}} = \sqrt{\sum_{i=1}^{N} |g_0(\mathbf{r}_i)|^2} = \frac{g_{\mathrm{CGT}}\mu_B}{2\hbar}\sqrt{\sum_{i=1}^{N} |B_1(\mathbf{r}_i)|^2} = \frac{g_{\mathrm{CGT}}\mu_B}{2\hbar} N_y \sqrt{\sum_{i=1}^{N}\left[(B_{x,i}^2 + B_{z,i}^2)\right]}. \tag{S4}$$

Here, μ_B is the Bohr magneton, N_y is the number of unit cells along the y-direction and g_{CGT} is the g-factor for CGT for which a value of 2.18[S10] is used. Note, we give the collective coupling strength for spin $1/2$ and for linear polarized microwaves[S3]. For the calculation of g_{eff} for the resonator loaded with 15 monolayers of CGT we extracted its lateral dimension from the AFM measurements (see Fig. S4 (g)) to 2 µm along the x-direction and 12 µm along the y-direction. The flake is assumed to lie directly on top of the superconducting wire without any gap in between. For these values the simulation yields $g_{\mathrm{eff}}/2\pi = 8.94\,\mathrm{MHz}$, which is about a factor 2.5 larger than the experimentally determined value of 3.61 MHz. The overestimation of the simulation most likely results from non-ideal conditions in the experiment. The corresponding flake lies at the top end of the resonators narrow wire section (see Fig. S5 (g)), where $\mathbf{B}_1$ is concentrated. The finite element simulations show that in this area the field strength is already declining, resulting in a reduced coupling strength. Further, AFM can overestimate the thickness of a flake slightly for when there is a gap between resonator surface and flake[S9]. The calculation also not includes the multiple peaks observed in the experiment, which - depending on their real nature - can distribute the magnon density over all resonant peaks. Nevertheless, we can use the simulation to estimate the signal reduction by scaling down the thickness of the flake to a single monolayer. Reducing the simulation to a single monolayer, while keeping the lateral dimensions, results in $g_{\mathrm{eff}}/2\pi = 2.33\,\mathrm{MHz}$, a reduction by a factor of 0.26.

IV. AFM MEASUREMENTS ON CGT FLAKES

After the transfer of the CGT flakes onto the individual resonators and after measuring FMR, we characterized the thickness of the flakes by AFM. Figure S5 shows a selection of height profile maps from the three resonator chips, including a height profile along the inductor wire of the resonator (blue line in the AFM profile images in Fig. S5). To extract the thickness we fit the steps in the height profile (red or green lines in the height profiles in Fig. S5). Note, the height values are relative values with an arbitrary offset. Figure S5 (g) shows the thinnest flake of this study, where the processed FMR data is shown in Fig. 4 in the main text.

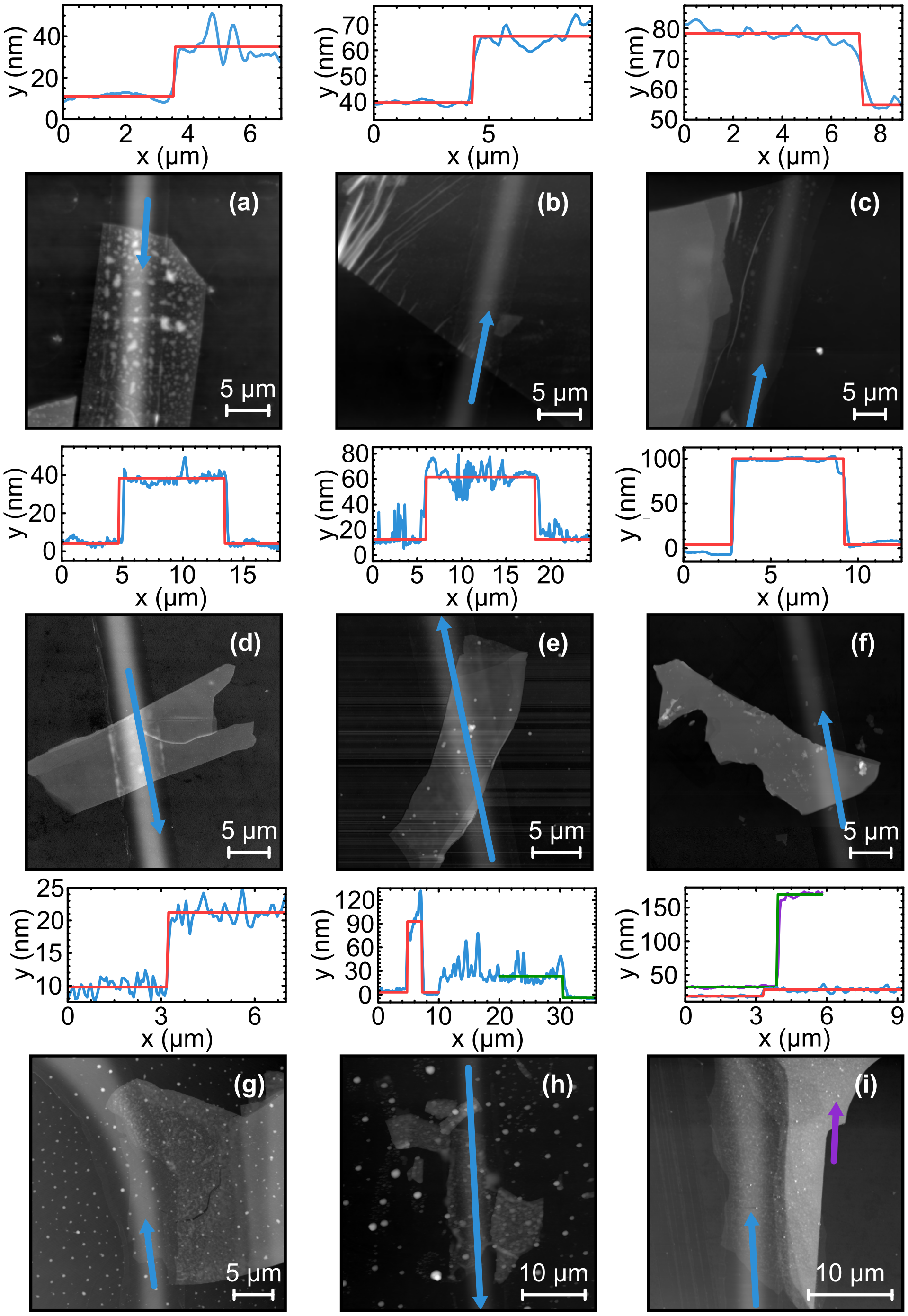

FIG. S5. **AFM measurements.** AFM profile images with respective height profile (above) along the resonator inductor wire (blue and purple lines in profile images, with the arrow indicating scan direction). **a** - **c** figures for resonator chip 1 (refer to Tab. I), having resonance frequencies with CGT of 17237 MHz, 17790 MHz and 16470 MHz, respectively. **d** - **f** figures for resonator chip 2 (refer to Tab. I), having resonance frequencies with CGT of 13244 MHz, 12063 MHz and 13620 MHz, respectively. **g** - **i** images for resonator chip 3 (refer to Tab. I), having resonance frequencies with CGT of 12314 MHz, 13272 MHz and 17054, MHz, respectively. The red and green solid lines are fits to the height profiles.

V. ANALYSIS AND ADDITIONAL FMR DATA

We analyze our experimental data, using the model functions (2) and (3) from the main text in a two-step semi-optimized fashion. The main intention for this approach is to minimize the number of free parameters in our model functions. In a first

coarse step, we match the collective coupling strength $g_{\mathrm{eff},k}$ to fit the experimental data, assume a constant separation between the individual magnon modes at $B_{\mathrm{FMR,k}}$ and the same magnon loss rate γ for all modes and determine the resonator loss rate κ_0 from the resonator transmission far detuned from the FMR with the CGT flakes. This results in 3 free parameters for the first stage of our analysis, the magnon loss rate γ, B_{FMR} of the main mode and the constant separation between the $B_{\mathrm{FMR,k}}$. After this first step we arrive at a best fit to the envelope of the experimental data, however with not matching amplitudes. In a consecutive second step, we manually optimize the $g_{\mathrm{eff},k}$ to arrive at a model in good agreement with ω_{res} and κ_{eff} (see dashed lines in Fig. S6).

Fig. S6 shows additional results from the corresponding FMR measurements performed on the in Fig. S5 showed resonators. As described in the main text, the measurements were performed at a temperature of 1.8 K and recording the microwave transmission $|S_{21}|^2$ as a function of the static magnetic field. Analyzing the microwave transmission by fitting a Fano resonance lineshape to it we extract the effective loss rate of the resonator, interacting with the CGT κ_{eff}. Figure S6 shows the resulting κ_{eff} as a function of the magnetic field. In general, the response of the CGT FMR is complex and varies for the different resonators. The resonance lineshape is not well described by just a single Lorentzian and requires multiple peaks to produce a good agreement. For some resonators, κ_{eff} exhibits obvious peaks, residing on a broader spectrum (see Fig. S6 (c), (f) and (i)). Together with the observation of well and clearly separated peaks for the resonator loaded with the thinnest CGT flake of 11 nm, we motivating the multiple peak analysis as presented in the main text. However, as the individual peaks are overlapping for the remainder of the resonators we only applied a qualitative analysis.

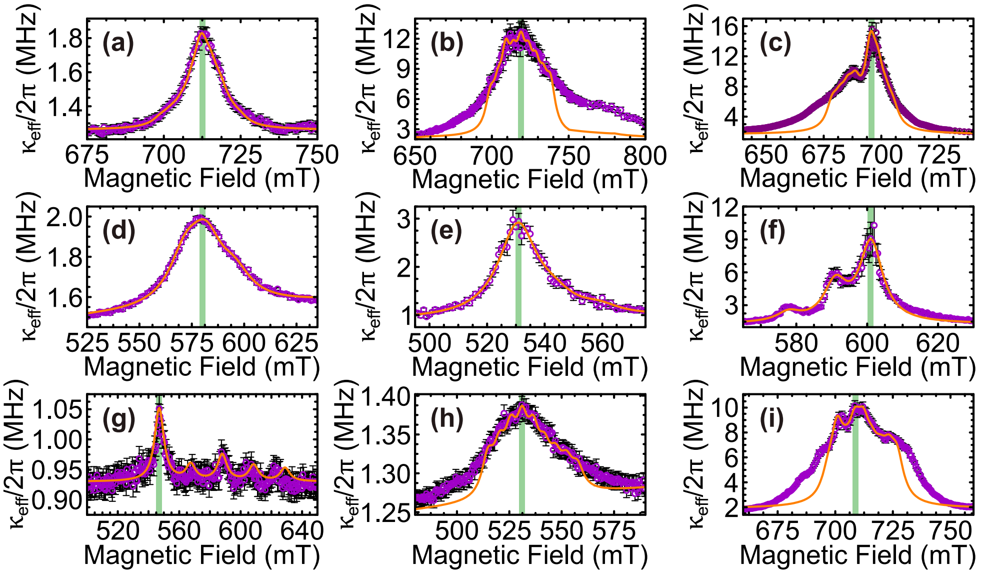

FIG. S6. **Additional data on magnon-photon coupling of CGT-resonator devices.** Results from FMR measurements with effective loss rate $\kappa_{\mathrm{eff}}/2\pi$ as a function of the static magnetic field. **a** - **c** results for resonator chip 1 (refer to Tab. I), having resonance frequencies with CGT of 17237 MHz, 17790 MHz and 16470 MHz, respectively. **d** - **f** results for resonator chip 2 (refer to Tab. I), having resonance frequencies with CGT of 13244 MHz, 12063 MHz and 13620 MHz, respectively. **g** - **i** results for resonator chip 3 (refer to Tab. I), having resonance frequencies with CGT of 12314 MHz, 13272 MHz and 11899 MHz, respectively. The orange solid lines are semi-optimized fits, as described in the main text. The errorbars in the figures represent the standard deviation from the Fano resonance lineshape fit to the respective resonator transmission.

Figure S7 shows the extracted collective coupling strength g_{eff} as a function of the square root of the FMR active volume. We define the active volume as the overlap of the oscillating magnetic field B_1 and the CGT flake lying on the resonator. The B_1 field distribution, discussed in Sec. III, is used to estimate the extend of the B_1 and is taken as 2 µm. From AFM measurements and microscope images we extract the thickness and lateral dimensions of the flakes to calculate the final active volume. As the collective coupling is proportional to the square root of the number of magnetic moments[S3], which are interacting with the resonator field, it follows that g_{eff} scales linearly with the square root of the active volume. This linear trend is highlighted by the orange solid line in Fig. S7. The majority of the extracted data follows this linear trend very well, corroborating our analysis. Only 3 data points deviate strongly from the rest of the data, which we attribute to significant inhomogeneities in the CGT-flakes, making the volume estimation inaccurate. These data points are highlighted in red in Fig. S7.

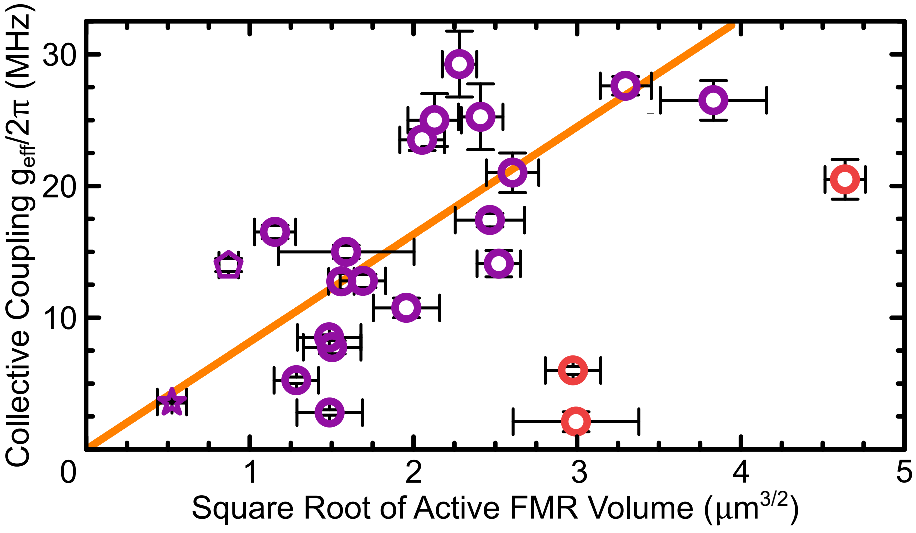

FIG. S7. **Scaling of the collective coupling.** Collective coupling strength g_{eff} as a function of the FMR active CGT-flake volume. The orange line highlights the linear trend of g_{eff} with increasing volume. The red symbols are regarded as outliers, as these flakes show inhomogeneities, leading to inaccurate volume estimations. The star symbol represents data from the thinnest flake (see data in Fig. 4 in the main text) and the pentagon symbol data from the 17 nm flake (see data in Fig. 2 in the main text) The errorbars give confidence values for the extracted values.

VI. MAGNETO-STATIC SPIN-WAVE DISPERSION IN THIN-FILM MAGNETS WITH PERPENDICULAR ANISOTROPY

Here we describe the spin-wave mode frequency in a thin-film magnet with perpendicular anisotropy along the film normal. We consider this at the magnetic-dipole limit where the wavelength is relatively large and the exchange interaction contribution to the spin-wave dispersion is neglected. Furthermore, standing spin-wave modes along the thickness direction are also ruled out since these modes only appear at much higher frequencies than the main mode, where we consistently observe additional peaks at both higher and lower frequencies from the main mode. The mode (angular) frequency (ω) for wavevector $k = 0$ when we apply a magnetic field B along one of the film plane directions can be given by Eq. 3d in Ref.[S11] as:

$$\left(\frac{\omega}{\gamma}\right)^2 = B\left(B + \mu_0 M_s - \frac{2K_u}{M_s}\right). \tag{S5}$$

Here, γ, M_s and K_u are the gyromagnetic ratio, saturation magnetization and the perpendicular anisotropy energy density, respectively. Note, that the total field within $\mu_0 M_s - \frac{2K_u}{M_s}$ is negative for perpendicularly-magnetized films which we consider in this section. Within the magnetic-dipole limit, the demagnetization term $\mu_0 M_s$ is modified for spin-waves with finite k, depending on the relative orientation between the M_s and k directions. Here we follow the expression given in Serga et al.[S12]. For pure backward volume magnetostatic modes where $k \parallel M_s$ (illustrated in Fig. S8), the mode frequency becomes:

$$\left(\frac{\omega_{\text{BVMSW}}}{\gamma}\right)^2 = B\left(B + \mu_0 M_s\left(\frac{1-e^{-kt}}{kt}\right) - \frac{2K_u}{M_s}\right), \tag{S6}$$

where t is the thickness of the magnet. Note, that this expression is only valid for the case where M_s is colinear to B, meaning that $|B| > |\mu_0 M_s - \frac{2K_u}{M_s}|$. To the limit of $k \to 0$, the term $(1-e^{-kt})/kt$ is reduced to unity, consistent to Eq. (S5). When k is nonzero, we can observe that ω_{BVMSW} becomes smaller than that for $k = 0$, exhibiting a negative group velocity for this spin-wave mode. As the opposite extreme where $k \perp M_s$ (illustrated in Fig. S8), the resonance frequency becomes larger than that for $k = 0$ and is called magneto-static surface spin-wave mode. The mode frequency expression for this mode is given by:

$$\left(\frac{\omega_{\text{MSSW}}}{\gamma}\right)^2 = B\left(B + \mu_0 M_s - \frac{2K_u}{M_s}\right) + \mu_0^2 M_s^2\left(1-e^{-2kt}\right). \tag{S7}$$

Here, $\mu_0^2 M_s^2\left(1-e^{-2kt}\right)$ is the spin-wave correction term which goes to zero for $k \to 0$ (hence consistent to Eq. (S5)) and becomes positive for $k > 0$, meaning that ω_{MSSW} becomes larger as soon as spin-waves gain momentum along this direction. We use these two expressions in an effort to explain the origin of the multiple peaks in our experiments. Figure S8 plots the calculated $\omega_{\text{BVMSW}}/2\pi$ and $\omega_{\text{MSSW}}/2\pi$ as a function of wavevector k. The range of wavevector is chosen such that the resulting resonance frequencies are within the same order of magnitude as the observed mode splittings in the experiment ($\propto 100\,\text{MHz}$).

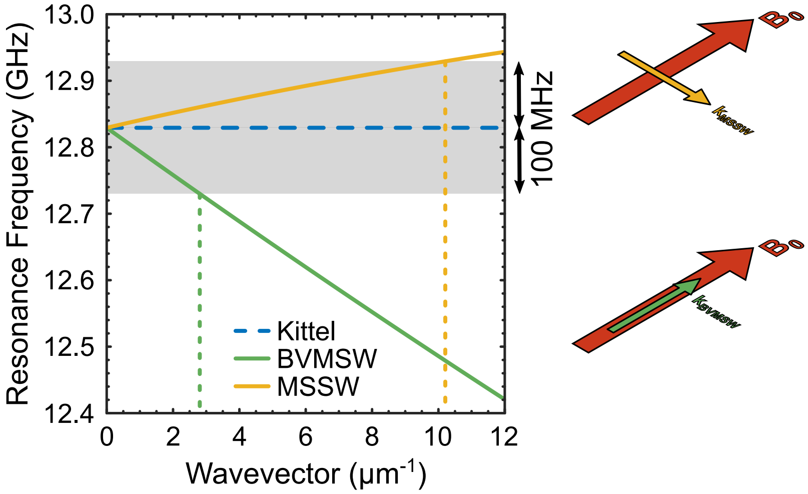

FIG. S8. **Spin-wave dispersion.** Spin-wave resonance frequency for BVMSW (green solid line) and MSSW (yellow solid line) as a function of wavevector. The dashed blue line is the resonance frequency of the $k = 0$ main mode. The parameters used are $B_0 = 598\,\mathrm{mT}$, $g_{\mathrm{CGT}} = 2.18$, $\mu_0 M_\mathrm{s} = 194.3\,\mathrm{mT}$ and $K_\mathrm{u} = 3.84 \times 10^4\,\mathrm{J/m^3}$ and a thickness of 17 nm. The grey area highlights a 100 MHz margin relative to the main mode, indicating the order of magnitude of the mode splitting observed in the experiment. The arrows on the right hand side illustrate the relative wavevector orientations of the BVMSW and MSSW spin-wave modes with respect to the static magnetic field.

The corresponding wavelength to a 100 MHz resonance offset to the main mode are about 2.2 µm and 620 nm for ω_{BVMSW} and ω_{MSSW}, respectively. These values are within a reasonable scale for our different lateral CGT flake dimensions under investigation. This suggests that spin-wave modes are likely the origin of the multiple resonance peaks observed.

The thinnest CGT flake shows, however, a deviation from this behaviour. We only observe modes at lower frequencies, which would indicate to BVMSW modes. Calculating the respective shortest wavelength results in 225 nm, which is significantly shorter than for the other devices. We assume that the placement and irregular shape are likely to cause this difference. First, this flake is placed at the very edge of the inductor wire, where the B_1 field strength is declining (see Fig. S3), reducing the FMR active area. Thickness steps can lead to a wavelength down-conversion[S13], however, with the overall irregular shape of the flake it is difficult to define a length scale for a standing spin wave mode.

VII. ATOMISTIC SPIN DYNAMICS SIMULATIONS OF FMR

To study the ferromagnetic resonance in CGT we perform atomistic spin dynamics simulations[S14,S15]. The magnetic Hamiltonian employed in the simulations is given by:

$$\mathscr{H} = -\frac{1}{2}\sum_{i,j}\mathbf{S}_i \mathscr{J}_{ij}\mathbf{S}_j - \sum_i D_i(\mathbf{S}_i\cdot\mathbf{e})^2 - \sum_i \mu_i \mathbf{S}_i\cdot(\mathbf{B_0}+\mathbf{B_1}) \tag{S8}$$

where i, j represent the atoms index, $\mathscr{J}_{ij}$ represents the exchange interaction tensor, D_i the uniaxial anisotropy, which for CGT is orientated out of plane ($\mathbf{e} = (0,0,1)$) and $\mathbf{B_0}$ the external static magnetic field applied in-plane during the ferromagnetic resonance simulations and $\mathbf{B_1} = B_1\sin(2\pi\nu t)$ the oscillating field applied perpendicular with respect to $\mathbf{B_0}$. The CGT system has been parameterized from first principle methods[S9], up to the third nearest neighbor intralayer and interlayer exchange. The exchange values have also been re-scaled by Gong et al.[S9] with a 0.72 factor to obtain the experimental T_C and multiplied by S^2 to match the magnetic Hamiltonian. The magnetic moment or Cr is considered 3.26 μ_B[S16] and the uniaxial anisotropy has a value of 0.05 meV as extracted from first principle methods[S9]. The parameters used in the simulations are given in Table II. FMR calculations have previously been employed for atomistic models, and can reproduced well the variation of linewidth with temperature, for example, in recording media systems[S17]. Hence, in the current simulations we use the same setup of frequency swept FMR[S17] and we obtain the spectra by performing a Fourier transform of the magnetisation component parallel to the oscillating field. Since these calculations are done close to 0K, no averaging is require to reduce the thermal noise. To excite the FMR mode, we apply a DC field in-plane of 0.9 T on x-direction and an AC field perpendicular to the DC field, on y-direction. The Fourier transform has been performed for the y-component of magnetisation for 5 ns after an initial 1 ns equilibration time. A thermal bath coupling has been chosen in agreement with the upper limit of the Gilbert damping observed in experiments.

The system size we performed FMR on is a 4-layer CGT system, with lateral size of 6.91 nm × 11.97 nm, periodic boundary conditions in xy and total of 1600 atoms. The small system size has been used to reduce the computational cost associated

Quantity	Symbol	quantity	units
Timestep	ts	0.1	fs
Thermal bath coupling	α	0.02	
Gyromagnetic ratio	γ_e	1.760859×10^{11}	rad $s^{-1}T^{-1}$
Magnetic moment	μ_B	3.26[S16]	μ_B
Uniaxial anisotropy	D_i	0.05[S9]	meV/link
Simulation temperature	T	0.001	K
Static magnetic field	B_0	0.9, 0.7	T
Oscillating magnetic field amplitude	B_0	0.001	T
FMR frequency	ν	varied	GHz
Intralayer exchange, NN	J_1	2.71[S9]	meV/link
Intralayer exchange, 2NN	J_2	- 0.058[S9]	meV/link
Intralayer exchange, 3NN	J_3	0.115[S9]	meV/link
Interlayer exchange, NN	J_1^z	-0.036[S9]	meV/link
Interlayer exchange, 2NN	J_2^z	0.086[S9]	meV/link
Interlayer exchange, 3NN	J_3^z	0.27[S9]	meV/link

TABLE II. Simulation parameters for FMR on CGT system

.

with FMR simulations. Experiments have showed modified g-factors due to photon-magnon coupling hence hereby we propose a simple model where the properties of the individual layers have been modified to include different gyromagnetic ratio, as illustrated in Fig. S9**a**.

We can define the resonance frequencies for each magnetic layer using the Kittel equation in the case of in-plane applied field with perpendicular anisotropy $B_{\perp \mathrm{u}}$:

$$\omega = \gamma\sqrt{B_0(B_0 - B_{\perp \mathrm{u}})} \tag{S9}$$

We next investigate the FMR signal for a few cases assuming the CGT monolayers at low or strong interlayer exchange couplings $J_z' = 0, 0.1\%, 10\%, 100\% J_z$, where J_z corresponds to the pristine interlayer exchange (Fig. S9**b-c**). In the low interlayer exchange regime ($J_z' = 0, 0.1\% J_z$), the CGT presents multiple peaks with each frequency corresponding to the layer dependent gyromagnetic ratio, γ - $\nu(\gamma_1) = 16.81\,\mathrm{GHz}$, $\nu(\gamma_2) = 25.22\,\mathrm{GHz}$, $\nu(\gamma_3) = 33.62\,\mathrm{GHz}$. At $J_z' = 0.1\% J_z'$ (Fig. S9**b**) we can still observe resonance peaks corresponding to each individual layer. However by increasing the exchange coupling to $10\% J_z'$ or higher (Fig. S9**c**) there is a single FMR peak indicating that the system behave coherently with all layers having the same FMR frequency. The single FMR frequency corresponds to the average magnetic properties of the CGT layers. Small variations of the resonance frequency as function of the inter-layer exchange coupling can be observed which these being correlated to the transition of the system from the multi-peaks regime to a coherent excitation. By calculating the damping of the highest resonance peaks from a Lorenzian fit, we reobtain the damping corresponding to the input thermal bath coupling, 0.02 with a relative tinny error $\sim 5\%$. Overall, the interlayer exchange coupling locks the dynamics of individual layers coherently together without allowing multiple frequencies at the FMR signal[S18].

[S1] A. E. Primenko, M. A. Osipov, and I. A. Rudnev, Technical Physics **62**, 1346 (2017).
[S2] L. H. Lee, T. P. Orlando, and W. G. Lyons, IEEE Transactions on Applied Superconductivity **4**, 41 (1994).
[S3] C. W. Zollitsch, K. Mueller, D. P. Franke, S. T. B. Goennenwein, M. S. Brandt, R. Gross, and H. Huebl, Applied Physics Letters **107**, 142105 (2015).
[S4] S. Weichselbaumer, P. Natzkin, C. W. Zollitsch, M. Weiler, R. Gross, and H. Huebl, Physical Review Applied **12**, 024021 (2019).
[S5] R. J. Schoelkopf and S. M. Girvin, Nature **451**, 664 (2008).
[S6] D. I. Schuster, A. A. Houck, J. A. Schreier, A. Wallraff, J. M. Gambetta, A. Blais, L. Frunzio, J. Majer, B. Johnson, M. H. Devoret, S. M. Girvin, and R. J. Schoelkopf, Nature **445**, 515 (2007).
[S7] Y. F. Li, W. Wang, W. Guo, C. Y. Gu, H. Y. Sun, L. He, J. Zhou, Z. B. Gu, Y. F. Nie, and X. Q. Pan, Physical Review B **98**, 125127 (2018).
[S8] Y. Sun, R. C. Xiao, G. T. Lin, R. R. Zhang, L. S. Ling, Z. W. Ma, X. Luo, W. J. Lu, Y. P. Sun, and Z. G. Sheng, Applied Physics Letters **112**, 072409 (2018).
[S9] C. Gong, L. Li, Z. Li, H. Ji, A. Stern, Y. Xia, T. Cao, W. Bao, C. Wang, Y. Wang, Z. Q. Qiu, R. J. Cava, S. G. Louie, J. Xia, and X. Zhang, Nature **546**, 265 (2017).
[S10] S. Khan, C. W. Zollitsch, D. M. Arroo, H. Cheng, I. Verzhbitskiy, A. Sud, Y. P. Feng, G. Eda, and H. Kurebayashi, Physical Review B **100**, 134437 (2019).
[S11] M. Farle, Reports on Progress in Physics **61**, 755 (1998).
[S12] A. A. Serga, A. V. Chumak, and B. Hillebrands, Journal of Physics D: Applied Physics **43**, 264002 (2010).
[S13] J. Stigloher, T. Taniguchi, M. Madami, M. Decker, H. S. Körner, T. Moriyama, G. Gubbiotti, T. Ono, and C. H. Back, Applied Physics Express **11**, 053002 (2018).
[S14] D. A. Wahab, M. Augustin, S. M. Valero, W. Kuang, S. Jenkins, E. Coronado, I. V. Grigorieva, I. J. Vera-Marun, E. Navarro-Moratalla, R. F. Evans, *et al.*, Advanced Materials **33**, 2004138 (2021).
[S15] A. Kartsev, M. Augustin, R. F. Evans, K. S. Novoselov, and E. J. G. Santos, npj Computational Materials **6**, 1 (2020).
[S16] I. A. Verzhbitskiy, H. Kurebayashi, H. Cheng, J. Zhou, S. Khan, Y. P. Feng, and G. Eda, Nature Electronics **3**, 460 (2020).

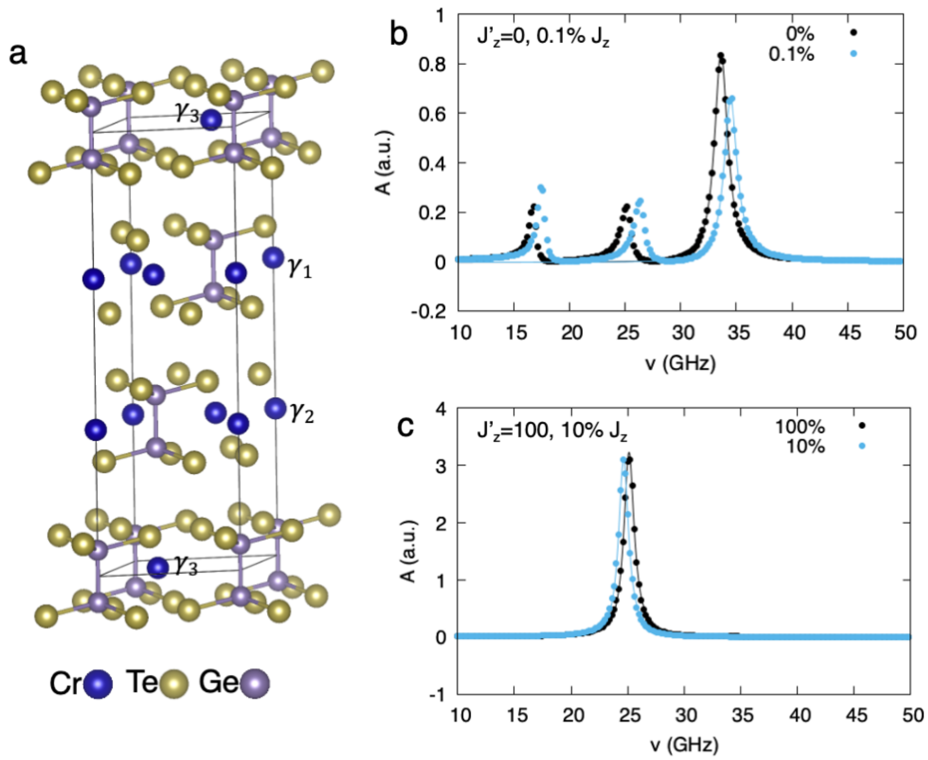

FIG. S9. **Atomistic simulations.** **a,** Schematic of the crystal structure of CGT with atoms defined by different colours. **b,** FMR spectra of 4 layer CGT where the layers are low interayer exchange coupled ($0, 0.1\%\ J_z'$, where J_z' is the pristine CGT interlayer exchange). **c,** Similar as **b,** but with the layers at a strong exchange coupling ($10\%, 100\%\ J_z'$). The solid lines in **b-c** represent a Lorenzian fit to the numerical data.

[S17]M. Strungaru, S. Ruta, R. F. Evans, and R. W. Chantrell, Physical Review Applied **14**, 014077 (2020).
[S18]Data inputs/plots utilised for Supplementary Figure S7 (atomistic simulations) can be found at the following GitHub repository.
